# Interaction of marijuana and alcohol on fatal motor vehicle crash risk: a case–control study

**DOI:** 10.1186/s40621-017-0105-z

**Published:** 2017-03-20

**Authors:** Stanford Chihuri, Guohua Li, Qixuan Chen

**Affiliations:** 1 Center for Injury Epidemiology and Prevention, Columbia University Medical Center, 622 West 168th St, PH5-505, New York, NY 10032 USA; 2 Department of Anesthesiology, College of Physicians and Surgeons, Columbia University, New York, NY USA; 3 Department of Epidemiology, Mailman School of Public Health, Columbia University, New York, NY USA; 4 Department of Biostatistics, Mailman School of Public Health, Columbia University, New York, NY USA

**Keywords:** Accidents, Traffic, Alcohol, Case–control studies, Drug interaction, Marijuana, Motor vehicles, Safety

## Abstract

**Background:**

Concurrent use of marijuana and alcohol in drivers is of increasing concern but its role in crash causation has not been well understood.

**Methods:**

Using a case–control design, we assessed the individual and joint effects of marijuana and alcohol use on fatal crash risk. Cases (*n* = 1944) were drivers fatally injured in motor vehicle crashes in the United States at specific times in 2006, 2007 and 2008. Controls (*n* = 7719) were drivers who participated in the 2007 National Roadside Survey of Alcohol and Drug Use by Drivers.

**Results:**

Overall, cases were significantly more likely than controls to test positive for marijuana (12.2% vs. 5.9%, *p* < 0.0001), alcohol (57.8% vs. 7.7%, *p* < 0.0001) and both marijuana and alcohol (8.9% vs. 0.8%, *p* < 0.0001). Compared to drivers testing negative for alcohol and marijuana, the adjusted odds ratios of fatal crash involvement were 16.33 [95% confidence interval (CI): 14.23, 18.75] for those testing positive for alcohol and negative for marijuana, 1.54 (95% CI: 1.16, 2.03) for those testing positive for marijuana and negative for alcohol, and 25.09 (95% CI: 17.97, 35.03) for those testing positive for both alcohol and marijuana.

**Conclusions:**

Alcohol use and marijuana use are each associated with significantly increased risks of fatal crash involvement. When alcohol and marijuana are used together, there exists a positive synergistic effect on fatal crash risk on the additive scale.

## Background

Driving under the influence of drugs (DUID) is a serious public safety concern in the United States and around the world (Brady & Li 2013; Romano & Voas 2011; Kaplan et al. 2006; Berning et al. 2015; Hartman & Huestis 2013; Walsh et al. 2005; Dubois et al. 2015). About one third of fatally injured drivers in the United States tested positive for nonalcohol drugs and 20% tested positive for two or more drugs (Brady & Li 2013; Romano & Voas 2011; Kaplan et al. 2006). The prevalence of non-alcohol drugs in weekend nighttime drivers is about 20% (Berning et al. 2015). Marijuana is the most frequently detected nonalcohol drug and the concurrent use of marijuana and alcohol is the most frequently detected poly-drug combination in the general driver population (Berning et al. 2015) and in fatally injured drivers (Walsh et al. 2005; Dubois et al. 2015; Li et al. 2013). Over the past decade, traffic fatalities involving nonalcohol drugs have increased markedly while fatalities involving alcohol have remained fairly stable (Brady & Li 2013; Li et al. 2013; Brady & Li 2014). Specifically, the prevalence of marijuana positivity in fatally injured drivers had tripled from 4% in 1999 to 12% in 2010 (Brady & Li 2014). Evidence from experimental studies has shown that marijuana impairs almost every aspect of performance associated with safe driving (Lenné et al. 2010; Bosker et al. 2012; Downey et al. 2013; Hartman et al. 2015) and recent epidemiologic studies have found that marijuana use may double the risk of car crash involvement (Li et al. 2012, Asbridge et al. 2012).

Since 1996, 28 states and Washington, DC have enacted legislation to decriminalize marijuana for medical use (NCSL 2016). Of these states, eight have further decriminalized possession of small amounts of marijuana for adult recreational use (NCSL 2016). Colorado has registered an increase in fatal motor vehicle crashes involving marijuana since legalizing marijuana for medical use in 2000 (Urfer et al. 2014) compared to insignificant changes in states without medical marijuana laws (Salomonsen-sautel et al. 2014).

Young adults may be at a particularly high risk from the increased availability and potency of marijuana (Brady & Li 2013; Cerda et al. 2012; Lynne-Landsman et al. 2013; Harper et al. 2012; Wall et al. 2011; O’Malley & Johnston 2007). Given the increasing prevalence of DUID (Brady & Li 2013; Berning et al. 2015; Dubois et al. 2015; Brady & Li 2014) and the increasing permissibility and accessibility of marijuana (NCSL 2016), it is urgent to better understand the role of marijuana and its interaction with alcohol in motor vehicle crashes. Previous research on the interaction between alcohol and marijuana has reported conflicting results, with some indicating the interaction effect to be possibly synergistic (i.e., the combined effect is more than the sum of the net effects of marijuana and alcohol) (Bates & Blakely 1999; Brault et al. 2004; Biecheler et al. 2008; Ramaekers et al. 2000; Ramaekers et al. 2004; Sewell et al. 2009; Sutton 1983; Perez-Reyes et al. 1988) or additive (i.e., the combined effect is equal to the sum of the net effects of marijuana and alcohol) (Ramaekers et al. 2004; Sewell et al. 2009; Bramness et al. 2010; Drummer et al. 2004; Chesher 1986; Kelly et al. 2004) and others suggesting no additive effect (Lamers & Ramaekers 2001; Liguori et al. 2002). Understanding the role of marijuana and its interaction with alcohol in motor vehicle crashes is important in order to develop targeted, effective public health interventions and prudent use of resources in mitigating the rising prevalence of DUID. In the present study, we use a population-based case–control study design and data from two national surveillance systems to assess the individual and joint effects of marijuana and alcohol use on the risk of fatal crash involvement.

## Methods

### Data sources

Data for this study came from two sources: 1) the Fatality Analysis Reporting System (FARS) and 2) the 2007 National Roadside Survey of Alcohol and Drug Use by Drivers (NRS). Both data systems are sponsored and maintained by the National Highway Traffic Safety Administration (Washington, DC). The FARS is a census of all crashes that occur on public roads in the United States and result in at least one death of an occupant or non-occupant within 30 days of the crash (National Highway Traffic Safety Administration 2012). This data repository includes detailed information on the people and vehicles involved as well as circumstances of the crash. Data elements include driver characteristics (e.g., age, sex, survival status, drug and alcohol test results, driving history within the past 3 years), vehicle characteristics (e.g., model, make, type, year, weight rating) and crash circumstances (e.g., date, time, light and atmospheric conditions) (National Highway Traffic Safety Administration 2012). Data are obtained from various state documents such as police accident reports, death certificates, coroner/medical examiner reports, state vehicle registration files, hospital medical reports and vital statistics (National Highway Traffic Safety Administration 2012). To ensure accuracy and completeness, several programs continuously monitor the data and each entry is automatically checked for acceptable range and consistency. Toxicological testing results for nonalcohol drugs are available for only about one-third of fatally injured drivers nationwide due to incomplete testing and reporting (NHTSA 2010, 2012). However, 12 states performed drug testing on more than 80% of fatally injured drivers during the study period.

The 2007 NRS was a national field survey based on voluntary and anonymous random stops of non-commercial drivers at 300 locations across the contiguous United States (Lacey et al. 2009). The sample was selected using a multistage random sampling method that include four levels; primary sampling units, police jurisdictions, survey locations and passing-by drivers (Lacey et al. 2009). Drivers at the designated study locations were randomly stopped and invited to participate in the survey. Verbally consented drivers responded to questions about their trip origin, destination, demographics, mileage and drinking behavior and provided an oral fluid sample for drug testing as well as a breath sample for alcohol testing. The 2007 NRS was conducted between 10 PM and midnight and 1 AM to 3 AM on Fridays and Saturdays and 9:30 AM to 11:30 AM and 1:30 PM to 3:30 PM on Fridays during July 20 through December 1, 2007. The overall response rate for the 2007 NRS was 70.7% (Lacey et al. 2009). For each driver, the final overall sampling probability was equivalent to the product of sampling probabilities at each of the four levels of sampling. The final sampling weight was computed as the inverse of the overall sampling probability, i.e., data from drivers who were unlikely to be interviewed based on the sampling procedure used were given more weight than data from drivers who were more likely to be interviewed (Lacey et al. 2009). Additional details of the sampling method and study protocol are described elsewhere (Lacey et al. 2009).

### Study design and study subjects

A population-based case–control design was used to assess the role of marijuana and the interaction effect of marijuana and alcohol on the risk of fatal crash involvement. Of the 3735 drivers in the sample, 1605 (42.9%) were excluded from the study due to missing drug testing results and 186 (4.9%) cases from Maryland, New Mexico and North Carolina were excluded because of concerns about the accuracy and reliability of drug testing data recorded in the FARS for these states (NHTSA 2012; SAMHSA 2010). Cases (*n* = 1944) were drivers with available drug testing results in the FARS who were involved in crashes at the same time of day and the same day of week as controls were interviewed (i.e., between 10 PM and midnight and 1 AM to 3 AM on Fridays and Saturdays and 9:30 AM to 11:30 AM and 1:30 PM to 3:30 PM on Fridays during July 20 through December 1 in 2006, 2007 and 2008 in the continental United States). Controls (*n* = 7719) were drivers with available drug testing results from oral fluid samples in the 2007 NRS. This study was deemed exempt from review under 45 CFR 46 by the Columbia University Medical Center Institutional Review Board (New York, NY).

### Drug testing assessments

Drug tests for cases were performed on blood and/or urine specimens using radioimmunoassay techniques and liquid/gas chromatography coupled with mass spectometry (Kaplan et al. 2006; Li et al. 2011). Overall, 1759 (90.5%) of the 1944 cases included in the analysis had at least one drug test based on blood specimens. For each case, the FARS records the presence of up to three nonalcohol drugs (Kaplan et al. 2006; National Highway Traffic Safety Administration 2012). In the presence of multiple drugs, nonalcohol drugs are recorded in the following order: narcotics, depressants, stimulants, marijuana and other illicit drugs including phencyclidine, anabolic steroid, inhalant and medications (Kaplan et al. 2006; National Highway Traffic Safety Administration 2012). If a drug and its metabolite were detected, only the parent drug was recorded. Cannabinoids was the drug class for marijuana products such as hashish, pot or weed (National Highway Traffic Safety Administration 2012). Blood alcohol concentrations (BACs) were recorded separately from nonalcohol drugs. Drug tests for controls were performed on oral fluid samples that were screened using enzyme-linked immunosorbent assay and then confirmed using liquid/gas chromatography–mass spectrometry (National Highway Traffic Safety Administration 2012). BACs for controls were determined from breath samples measured using a preliminary breath test device (National Highway Traffic Safety Administration 2012; Lacey et al. 2009).

### Statistical analysis

The associations of alcohol and marijuana use with the risk of fatal crash involvement were measured by estimated odds ratios (ORs) and 95% confidence intervals (95% CIs) with drivers who tested negative as the reference group. Crude ORs and 95% CIs were computed for driver age, sex, marijuana testing result and blood alcohol concentration. BAC was measured in grams per deciliter and a value of 0.01 g/dL or greater was considered positive. BAC was categorized into 3 levels; 0, 0.01–0.07 and ≥0.08 g/dL according to current per se laws in the United States. Separate and joint effects of marijuana and BAC levels were assessed, with drivers testing negative for marijuana and alcohol as the reference group. Interaction of marijuana and alcohol was first examined as a departure from multiplicativity, i.e., whether the joint effect of alcohol and marijuana differs from the multiplicative model expected on the basis of their separate net effects adjusting for demographic variables. To assess the presence, magnitude and direction of interaction on the multiplicative scale, the estimated adjusted odds ratios from the logistic regression models were substituted into the following equation (Rothman 1986; VanderWeele 2014):

$$ \mathsf{O}{\mathsf{R}}_{\mathsf{marijuana}+\mathsf{alcohol}}/\left[\left(\mathsf{O}{\mathsf{R}}_{\mathsf{marijuana}}\right)*\left(\mathsf{O}{\mathsf{R}}_{\mathsf{alcohol}}\right)\right] $$

where a value >1 implies presence of positive interaction, a value < 1 implies presence of negative interaction and a value of 1 implies absence of interaction. Interaction was also tested as a departure from additivity, i.e., whether the joint effects of alcohol and marijuana were in excess of the sum of their individual effects. Additive interaction was assessed based on three measures; the relative excess risk due to interaction (RERI), the attributable proportion due to interaction (API) and the synergy index (S) as shown below (Rothman 1986; Knol et al. 2012; VanderWeele & Knol 2014; Andersson et al. 2005):

$$ \mathsf{RERI} = \mathsf{O}{\mathsf{R}}_{\mathsf{marijuana}+\mathsf{alcohol}}\hbox{--} \mathsf{O}{\mathsf{R}}_{\mathsf{marijuana}}\hbox{--}\ \mathsf{O}{\mathsf{R}}_{\mathsf{alcohol}} + \mathsf{1} $$

$$ \mathsf{A}\mathsf{P}\mathsf{I} = \mathsf{RERI}/\mathsf{O}{\mathsf{R}}_{\mathsf{marijuana}+\mathsf{alcohol}} $$

$$ \mathsf{S} = \left[\mathsf{O}{\mathsf{R}}_{\mathsf{marijuana}+\mathsf{alcohol}}\hbox{--} \mathsf{1}\left]/\right[\left(\mathsf{O}{\mathsf{R}}_{\mathsf{marijuana}}\hbox{--} \mathsf{1}\right) + \left(\mathsf{O}{\mathsf{R}}_{\mathsf{alcohol}}\hbox{--} \mathsf{1}\right)\right] $$

where RERI = 0, API = 1 and S = 1 denotes absence of interaction. Estimates for RERI and 95% CI from the delta method were computed using an approach suggested by VanderWeele and Knol (VanderWeele & Knol 2014). Lastly, we assessed marijuana as an effect modifier (VanderWeele 2009), i.e., whether the effect of BAC on the risk of crash involvement was homogenous between marijuana positive and negative drivers. The Tarone adjusted Breslow Day test (Breslow & Day 1980; Tarone 1985) was used to assess homogeneity between marijuana strata. To ensure that our findings were robust, we performed three separate sensitivity analyses: first, by including only cases from the 12 states that tested more than 80% of fatally injured drivers during the study period; second, by analyzing weighted data accounting for the complex sampling design among controls (Lacey et al. 2009); and third, by conducting multiple imputation for missing marijuana testing results among cases to assess the potential bias from the missing marijuana data. The missing marijuana testing data were imputed using a logistic regression model with covariates including age, sex, license status, previous incidents, year, day and time of crash, use of restraint, police reported drug use, vehicle role in the crash, and region. Ten imputations were generated. All analyses were performed using SAS 9.4 (SAS Institute Inc., Cary, NC). The multiple imputation was performed using the SAS callable software IVEware (Survey Research Center, Institute for Social Research, University of Michigan, Ann Arbor, MI).

## Results

Compared to the excluded drivers, those included as cases (*n* = 1944) were younger (mean age =36.8 (standard deviation, 16.8) years vs. 40.1 (standard deviation, 19.7) years), more likely to be involved in nighttime crashes (70.1% vs. 63.3%, *P* < 0.0001), more likely to be male (82.8% vs. 76.9%, *P* < 0.0001), and more likely to be involved in a crash in the previous 3 years (12.8% vs. 11.0%, *P* = 0.01).

Overall, 12.2% of the 1944 cases and 5.9% of the 7719 controls tested positive for marijuana, yielding a crude OR of 2.21 (95% CI: 1.87, 2.60). Positive BACs (BAC ≥ 0.01 g/dL) were present in 57.8% of cases and 7.7% of controls, yielding a crude OR of 16.42 (95% CI: 14.52, 18.57). As expected, the risk of fatal crash involvement increased with BAC levels from a crude OR of 2.65 (95% CI: 2.16, 3.25) for 0.01–0.07 g/dL to 63.14 (95% CI: 52.01, 76.65) for ≥0.08 g/dl (Table 1). Male drivers and drivers aged 16–39 years as well as those aged 65 years and older were at significantly increased risk of fatal crash involvement (Table 1).

**Table 1:** Crude odds ratios and 95% confidence intervals of fatal crash involvement according to driver age, sex, alcohol and marijuana testing results in the continental United States, selected time periods on Fridays and Saturdays, July 20 through December 1, 2006, 2007 and 2008

Characteristic	% of cases^a^ (*n* = 1944)	% of controls^b^ (*n* = 7719)	Crude OR	95% CI
Age (years)				
16–24	29.7	28.7	1.20	1.05, 1.36
25–39	33.2	32.0	1.20	1.06, 1.36
40–64	29.5	34.2	1.00	
≥ 65	7.6	5.1	1.70	1.38, 2.10
Sex				
Female	17.3	39.8	1.00	
Male	82.7	60.2	3.16	2.79, 3.58
Blood alcohol concentration (g/dL)				
0.00	42.2	92.3	1.00	
0.01–0.07	7.2	5.9	2.65	2.16, 3.25
≥ 0.08	50.6	1.8	63.14	52.01, 76.65
Marijuana				
Negative	87.8	94.1	1.00	
Positive	12.2	5.9	2.21	1.87, 2.60

*CI* Confidence Interval, *OR* Odds Ratio

^a^There were 23 cases with missing data on blood alcohol concentration

^b^There were 93 controls with missing data on age, 21 controls with missing data on sex and 7 controls with missing data on blood alcohol concentration

Relative to drivers who tested negative for both alcohol and marijuana, the estimated odds of fatal crash involvement increased 16 fold for those testing positive for alcohol and negative for marijuana, 1.5 fold for those testing negative for alcohol and positive for marijuana positive, and over 25 fold for those testing positive for both alcohol and marijuana (Table 2).

**Table 2:** Estimated odds ratios and 95% confidence intervals of fatal crash involvement according to driver alcohol and marijuana testing results in the continental United States, selected time periods on Fridays and Saturdays, July 20 through December 1, 2006, 2007 and 2008

Testing Result					
Marijuana	Alcohol	Crude OR	95% CI	^a^Adjusted OR	95% CI
Negative	Negative	1.00	Reference	1.00	Reference
Negative	Positive	15.93	13.99, 18.14	16.33	14.23, 18.75
Positive	Negative	1.46	1.11, 1.92	1.54	1.16, 2.03
Positive	Positive	23.31	16.92, 32.12	25.09	17.97, 35.03

*CI* Confidence Interval, *OR* Odds Ratio

^a^Adjusted for age, sex and region

The joint odds ratio of marijuana at each BAC level (OR_0.01–0.07g/dL_ = 4.38; OR_≥0.08g/dL_ =95.26) was equivalent to the product of the individual marijuana and alcohol odds ratios at each BAC level (OR_0.01–0.07g/dL_ = 1.56*2.81; OR_≥0.08g/dL_ =1.56*61.11), indicating the absence of interaction on the multiplicative scale (Table 3). However, significant interaction was present on the additive scale for combined BAC levels (RERI = 2.94, 95% CI: 0.60, 5.28) and for the separate BAC levels [(RERI_0.07g/dL_ =1.01, RERI_≥0.08g/dL_ = 32.59), (API_0.07g/Dl_ = 0.23, API_≥0.08g/dL_ =0.34), (S_≥0.08g/dL_ =1.43, S_≥0.08g/dL_ = 1.55)].

**Table 3:** Estimated odds ratios and 95% confidence intervals of fatal crash involvement according to driver BAC level and marijuana testing results in the continental United States, selected time periods on Fridays and Saturdays, July 20 through December 1, 2006, 2007 and 2008

Testing Result					
Marijuanal	BAC leve	Crude OR	95% CI	^a^Adjusted OR	95% CI
Negative	0	1.00	Reference	1.00	Reference
Positive	0	1.46	1.11, 1.92	1.56	1.18, 2.06
Negative	0.01–0.07	2.72	2.20, 3.38	2.81	2.25, 3.50
Positive	0.01–0.07	3.99	2.77, 5.73	4.38	3.01, 6.37
Negative	≥0.08	59.73	48.72, 73.23	61.11	49.50, 75.46
Positive	≥0.08	87.42	61.18, 124.90	95.26	65.75, 138.02

*BAC* Blood Alcohol Content, g/dL, *CI* Confidence Interval *OR* Odds Ratio

^a^Adjusted for age, sex and region

The estimated odds ratios of fatal crash involvement increased with BAC level in similar degrees between drivers testing positive for marijuana and those testing negative for marijuana (Table 4) and there was no significant heterogeneity in the estimated odds ratios associated with BAC level between the marijuana strata (Breslow Day Chi-square = 2.49, *p* = 0.288). Estimated odds ratios from all three sensitivity analyses were consistent with those derived from the actual drug testing data (Appendix). Results from all sensitivity analyses showed a significant positive interaction on the additive scale [(RERI_1_ = 4.46 95% CI: 0.98, 7.95; RERI_2_ = 2.90 95% CI: 0.60, 5.27; RERI_3_ = 2.77 95% CI: 2.11, 3.42)], but no interaction on the multiplicative scale.

**Table 4:** Estimated odds ratios and 95% confidence intervals of fatal crash involvement according to driver BAC level stratified by marijuana status in the continental United States, selected time periods on Fridays and Saturdays, July 20 through December 1, 2006, 2007 and 2008

Testing Result	Marijuana Negative		Marijuana Positive	
BAC level	^a^Adjusted OR	95% CI	^a^Adjusted OR	95% CI
0	1.00	Reference	1.00	Reference
0.01–0.07	2.81	2.25, 3.51	1.81	0.93, 3.50
≥0.08	60.96	49.37, 75.25	82.12	41.73,161.61

*BAC* Blood Alcohol Content, g/dL, *CI* Confidence Interval *OR* Odds Ratio

^a^Adjusted for age, sex and region

## Discussion

Results of this study indicate that marijuana use is associated with a significantly increased risk of involvement in fatal motor vehicle crashes, as reported in recent epidemiological studies (Li et al. 2013; Li et al. 2012; Asbridge et al. 2012). The combined effect of marijuana and alcohol was greater than the sum of the net effects of the two substances, which suggests that each substance plays a significant role in drivers’ involvement in fatal motor vehicle crashes. All three measures (RERI, API and S) confirmed the presence of significant positive interaction between alcohol and marijuana on fatal crash risk on the additive scale. Assuming positive monotonicity, i.e., both exposures (marijuana and alcohol) are never protective, the positive additive interaction suffices for mechanistic or sufficient cause interaction (Tarone 1985; VanderWeele & Robins 2007), which is indicative of synergism. These findings are important because the possible interaction between marijuana and alcohol on driving safety has long been a cause of concern (Sutton 1983; Gjerde & Kinn 1991; Stramer & Bird 1984) and recent toxicological data have shown that more that 20% of drivers fatally injured in motor vehicle crashes test positive for two or more drugs (Brady & Li 2013; Romano & Voas 2011; Kaplan et al. 2006) with alcohol and marijuana being the most common combination (Brady & Li 2013; Walsh et al. 2005).

Our results are consistent with previous experimental and epidemiological studies that have reported additive effects of alcohol and marijuana on driving performance and crash risk (Dubois et al. 2015; Bates & Blakely 1999; Sewell et al. 2009; Doty et al. 1992; Belgrave et al. 1979; Drummer et al. 2004; Chesher 1986) and possibly synergistic (Brault et al. 2004; Biecheler et al. 2008; Sutton 1983; Perez-Reyes et al. 1988) effects. For instance, a recent case–control study found that, relative to drivers using neither alcohol nor marijuana, drivers who tested positive for both alcohol (BAC ≤ 0.08 g/dL) and marijuana had up to 128% increased odds of committing an unsafe driver action compared to 16% for those using marijuana alone and 117% for those using alcohol (BAC ≤ 0.08 g/dL) alone (Dubois et al. 2015). Previous studies that reported modest or no interaction between marijuana and alcohol (Lamers & Ramaekers 2001; Liguori et al. 2002) analyzed very small study samples and assessed the effect of drugs on isolated experimental dependent measures such as reaction time (Liguori et al. 2002) or visual search at intersections (Lamers & Ramaekers 2001) instead of crash involvement in real traffic situations. In addition to the large sample size, this study was based on data from 46 states, including 10 out of 13 states that had legalized marijuana for medical use as of 2008. Therefore, the findings of this study could be considered more representative of the United States than those reported in previous studies.

The present study provides valuable evidence for understanding the interaction of drugs and driving safety. Many models in epidemiologic studies are inherently multiplicative and tend to evaluate interaction on the multiplicative scale only (Knol et al. 2009). This study assessed interaction on both multiplicative and additive scales. There has been debate in the epidemiologic literature as to which scale is better for assessing interactions (Blot & Day 1979; Saracci 1980; Rothman et al. 1980). Some epidemiologists have pointed out the potential limitations of using statistical interaction to draw conclusions about biological interaction (Siemiatycki & Thomas 1981; Thompson 1991; Cordell 2002). In this study, we assumed three sufficient causes of the outcome (fatal crash involvement), with marijuana, alcohol and both marijuana and alcohol as the three disparate causal mechanisms. Biological interaction was assessed where both marijuana and alcohol are single component causes in a combined causal mechanism. The additive model underpins the methods for assessing biological interaction (Rothman et al. 1980; Rothman 1976) and the notion of biological synergism (Rothman 1976), which may be difficult to interpret from multiplicative models. Although in some cases the multiplicative scale may naturally correspond to interaction mechanisms (Siemiatycki & Thomas 1981), the additive scale is of greater public health importance (VanderWeele 2011) because it allows researchers to discern the effect difference among subgroups.

The risk of fatal crash involvement associated with alcohol use appears to be homogenous between marijuana strata, indicating that marijuana is not a significant effect modifier in the BAC-fatal crash risk relationship. The homogeneity test results corroborate the absence of interaction on the multiplicative scale. At low doses, marijuana and alcohol may be mostly associated with impairment of low level operational driving skills (Lamers & Ramaekers 2001; Liguori et al. 2002) with gradual impairment of higher-level driving skills such as hazard perception, risk management and self-control as the doses increase. With the rising prevalence of marijuana use, potency, social tolerance, early age of onset and availability (Brady & Li 2014; Cerda et al. 2012; Lynne-Landsman et al. 2013; O’Malley & Johnston 2007), public health efforts should focus on strengthening and expanding drug testing as well as intervention programs for drivers. Although it is illegal to drive under the influence of alcohol and drugs in all states, almost all of the one million registered medical marijuana users are from states with older and less regulated programs that have minimal physician or state oversight (Williams et al. 2016). Therefore it is urgent to develop intervention programs to reduce the unintended health consequences of state laws legalizing marijuana for medical and/or recreational use, such as injuries and fatalities resulting from DUID.

This study has several notable limitations. First, drug testing protocols vary from state to state (Asbridge 2014). Some states have low testing rates, which may introduce selection bias. However, only subjects with known drug testing results were included in this study and results from the sensitivity analyses, including multiply imputed drug testing results for excluded drivers, were consistent with those from the actual testing data. Second, there are additional possible causal components for crash involvement that are not measured in the study, such as driver’s health status, comorbidity, medication use and weather condition and other environmental factors. Third, a positive test for marijuana indicates marijuana use but not necessarily marijuana-induced impairment as marijuana metabolites could remain in the blood for weeks after use (Voas et al. 2011; Skopp & Potsch 2008). Finally, marijuana tests were based on blood and urine specimens for cases and oral fluids for controls. The oral fluid testing method is widely used for detecting recent use of drugs such as marijuana, with reported sensitivity of 86–90% and specificity of 75–77% (Bosker & Huestis 2009; Desrosiers et al. 2012).

## Conclusions

Alcohol and marijuana are each associated with heightened risk of fatal crash involvement. When alcohol and marijuana are used together, there exists a positive synergistic effect on the risk of fatal crash involvement on the additive scale. These results suggest that the combined effects of alcohol and marijuana on fatal crash risk are significantly greater than the sum of their separate effects. With the rising prevalence of marijuana use, potency, social tolerance, early age of onset and availability, drug testing in drivers should be strengthened and expanded and effective intervention programs to reduce DUID and its adverse consequences should be implemented. Our findings imply that countermeasures simultaneously targeting alcohol-impaired driving and DUID might be more effective in improving driving safety than interventions targeting alcohol-impaired driving and DUID in isolation. Epidemiologic research aimed at quantifying the dose–response effect of marijuana on crash risk is urgently needed.

## Appendix

**Table 5:** Estimated odds ratios and 95% confidence intervals of fatal crash involvement according to driver BAC level and marijuana testing results in states that tested at least 80% of All fatally injured drivers, selected time periods on Fridays and Saturdays, July 20 through December 1, 2006, 2007 and 2008

Testing Result					
Marijuana	BAC level	Crude OR	95% CI	^a^Adjusted OR	95% CI
Negative	0	1.00	Reference	1.00	Reference
Positive	0	1.58	1.07, 2.35	1.76	1.17, 2.66
Negative	0.01-0.07	2.59	1.88, 3.55	2.66	1.91, 3.70
Positive	0.01-0.07	4.10	2.41, 6.96	4.69	2.69, 8.19
Negative	≥0.08	57.99	45.80, 73.43	58.33	44.91, 75.75
Positive	≥0.08	91.75	56.41, 149.22	102.94	61.13, 173.32

*BAC* Blood Alcohol Content, g/dL, *CI* Confidence Interval, *OR* Odds Ratio

^a^Adjusted for age, sex and region

**Table 6:** Estimated odds ratios and 95% confidence intervals of fatal crash involvement according to weighted driver BAC level and marijuana testing results, selected time periods on Fridays and Saturdays, July 20 through December 1, 2006, 2007 and 2008

Testing Result					
Marijuana	BAC level	Crude OR	95% CI	^a^Adjusted OR	95% CI
Negative	0	1.00	Reference	1.00	Reference
Positive	0	1.44	1.03, 2.00	1.52	1.10, 2.08
Negative	0.01-0.07	2.80	2.12, 3.70	3.01	2.31, 3.93
Positive	0.01-0.07	4.02	2.57, 6.30	4.56	2.96, 7.04
Negative	≥0.08	63.57	45.05, 89.69	66.50	47.51, 93.08
Positive	≥0.08	91.34	55.26, 150.97	100.78	61.78, 164.37

BAC, Blood Alcohol Content, g/dL; CI, Confidence Interval; OR, Odds Ratio

^a^Adjusted for age, sex and region

**Table 7:** Estimated odds ratios and 95% confidence intervals of fatal crash involvement according to driver BAC level and multiply imputed marijuana testing results, selected time periods on Fridays and Saturdays, July 20 through December 1, 2006, 2007 and 2008

Testing Result					
Marijuana	BAC level	Crude OR	95% CI	^a^Adjusted OR	95% CI
Negative	0	1.00	Reference	1.00	Reference
Positive	0	1.73	1.35, 2.21	1.84	1.43, 2.36
Negative	0.01-0.07	2.06	1.70, 2.50	2.34	1.90, 2.89
Positive	0.01-0.07	2.07	1.18, 3.62	2.49	1.40, 4.40
Negative	≥0.08	53.58	44.10, 65.11	60.43	49.37, 73.98
Positive	≥0.08	88.91	49.04, 161.18	101.93	56.70, 183.25

*BAC* Blood Alcohol Content, g/dL, *CI* Confidence Interval, *OR* Odds Ratio

^a^Adjusted for age, sex and region

## Abbreviations

AP: Attributable proportion due to interaction
BAC: Blood alcohol concentration
CI: Confidence interval
DC: District of Columbia
FARS: Fatality Analysis Reporting System
NHTSA: National Highway Traffic Safety Administration
NRS: National Roadside Survey of Alcohol and Drug Use by Drivers
OR: Odds ratio
RERI: Relative excess risk due to interaction
S: The synergy index
